# Community dynamics during de novo colonization of the nascent peri-implant sulcus

**DOI:** 10.1038/s41368-025-00367-7

**Published:** 2025-04-29

**Authors:** Tamires Pereira Dutra, Nicolas Robitaille, Khaled Altabtbaei, Shareef M. Dabdoub, Purnima S. Kumar

**Affiliations:** 1 Department of Periodontics and Oral Medicine, University of Michigan - School of Dentistry. 1011 N. University Ave, Ann Arbor, MI USA; 2 Division of Periodontology, College of Dentistry, The Ohio State University, Columbus, OH USA; 3 Faculty of Medicine and Dentistry, School of Dentistry, University of Alberta, Edmonton, AB Canada; 4 Department of Periodontics, The University of Iowa College of Dentistry, Iowa City, IA USA; 5 Division of Biostatistics and Computational Biology, The University of Iowa College of Dentistry, Iowa City, IA USA

**Keywords:** Peri-implantitis, Metagenomics, Microbial ecology

## Abstract

Dental implants have restored masticatory function to over 100 000 000 individuals, yet almost 1 000 000 implants fail each year due to peri-implantitis, a disease triggered by peri-implant microbial dysbiosis. Our ability to prevent and treat peri-implantitis is hampered by a paucity of knowledge of how these biomes are acquired and the factors that engender normobiosis. Therefore, we combined a 3-month interventional study of 15 systemically and periodontally healthy adults with whole genome sequencing, fine-scale enumeration and graph theoretics to interrogate colonization dynamics in the pristine peri-implant sulcus. We discovered that colonization trajectories of implants differ substantially from adjoining teeth in acquisition of new members and development of functional synergies. Source-tracking algorithms revealed that this niche is initially seeded by bacteria trapped within the coverscrew chamber during implant placement. These pioneer species stably colonize the microbiome and exert a sustained influence on the ecosystem by serving as anchors of influential hubs and by providing functions that enable cell replication and biofilm maturation. Unlike the periodontal microbiome, recruitment of new members to the peri-implant community occurs on nepotistic principles. Maturation is accompanied by a progressive increase in anaerobiosis, however, the predominant functionalities are oxygen-dependent over the 12-weeks. The peri-implant community is easily perturbed following crown placement, but demonstrates remarkable resilience; returning to pre-perturbation states within three weeks. This study highlights important differences in the development of the periodontal and peri-implant ecosystems, and signposts the importance of placing implants in periodontally healthy individuals or following the successful resolution of periodontal disease.

## Introduction

Nearly 60% of Americans over 50 years have less than 21 teeth; the minimum required for optimal function.^1^ Partial or complete edentulism places an enormous burden on an individual’s physical (oral and general health) and psychological well-being,^2,3^ as well as financial cost.^3^ In 1978, the titanium root-form implant was introduced;^4^ and by the 1990s, two novel biofilm-induced inflammatory diseases had been documented: peri-implant mucositis and peri-implantitis.^5^ Peri-implantitis occurs in 1/10^th^ of all implants, and peri-implant mucositis in 60% of implants.^6^ The onset and course of peri-implantitis is highly unpredictable; and an implant can be lost within a few months of diagnosis, leading to large soft-tissue and bone deficits that require significant reconstruction.^7^ Evidence implicates history of periodontal disease, poor plaque control and lack of compliance with maintenance therapy in increasing the risk for peri-implantitis; and the common thread linking these factors is that they all contribute to creating and perpetuating dysbiotic implant microbiomes.^8–11^

In order to elucidate the role of microbial dysbiosis in the onset and progression of peri-implant diseases, it is important to define the characteristics of a normobiotic community. While natural teeth have evolved to recognize microbial patterns and establish a homeostatic relationship with the resident community, implants lack a similar evolutionary heritage. Indeed, it is now established that the topography and surface characteristics of dental and other biomedical implants alter the natural environment, and that the peri-implant microbiome differs substantially from subgingival microbiome on even adjoining teeth.^12^ Hence, understanding the dynamics and structure of the peri-implant microbiome during de novo colonization will improve our ability to prevent, diagnose, and monitor peri-implant diseases.

Therefore, we aimed to investigate the sequence of bacterial colonization around dental implants in humans from initial exposure to the oral environment (uncovery) until after functional loading with the placement of a permanent crown by combining a longitudinal clinical study design with whole-genome shotgun sequencing, fine-scale enumeration and graph theoretics.

## Results

15 subjects who gave informed consent and were initially enrolled completed all visits with no dropouts. Table 1 summarizes the demographic and clinical baseline information of the patients included. In summary, the study population included 8 females and 7 males; their average age of 59.53 ( ± 14.13) years old. Twelve subjects were Caucasians, two were of Hispanic origin, and one identified as African-American. All implants installed were TSV with microtextured surface (Zimmer Biomet®, Palm Beach Gardens, FL), with 73.3% placed in the maxilla. No incidents of clinical or mechanical complications were identified through the study follow-up.

**Table 1:** Baseline sociodemographic and clinical characteristics of the study population

Items	No. of patients (%) or parameter value
*Age (Years* ± *SD)*	59.53 ± 14.13
*Gender (%F)*	53.55%
*Race – (%Caucasion)*	80%
*Implant Location - (%upper-arch)*	73.3%
*Plaque index (mean* *±* *SD)*	0.03 ± 0.12
*Gingival index (mean* *±* *SD)*	1.43 ± 0.86
*Bleeding on probing (%Yes)*	93%

Whole genome shotgun sequencing generated 3.7 billion paired-end sequences (250 bp). These sequences represented 17 777 functionally annotated microbial genes (based on KEGG classification), 548 species-level, and 2 109 strain-level sequence identifications (based on eHOMD classification).

### The coverscrew chamber is the preponderant source of the peri-implant microbial community at 24 hours

Over 500 species were identified in varying abundances in the coverscrew chamber, indicating that this environment hosts a rich and diverse community. *Streptococcus mitis* (19.123%), *Enterococcus casseliflavus* (7.20%), *Cutibacterium acnes* (5.58%), *Haemophilus parainfluenzae* (3.44%), and *Saccharibacteria (TM7) [G-1] bacterium HMT 348* (3.40%), were the most abundant species (Supplementary Table S1). Of all 548 species identified, 240 were present in 100% of the coverscrew chambers (core microbiome), indicating that almost half (43.87%) of the microbiome is conserved among all implants at uncovery. Furthermore, these core species contributed to 78% of the microbial abundance in the coverscrew chamber, indicating that most of the heterogeneity in the coverscrew chamber microbiome is attributable to rare taxa.

**Fig. 1:**
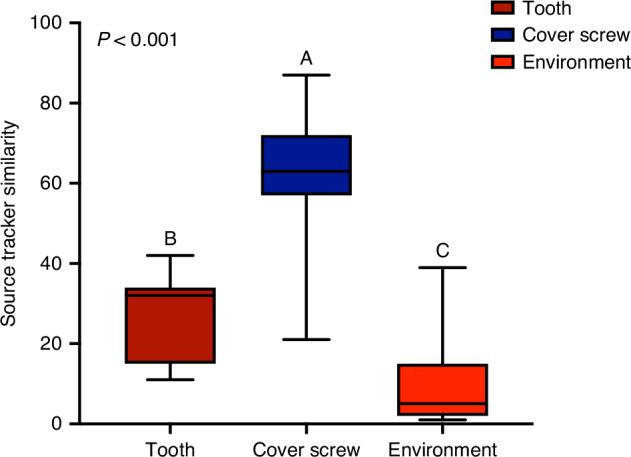
The coverscrew chamber as a source of pioneer species of the peri-implant microbiome Fig. 1 is the sources of the pioneer species in the pristine implant sulcus. The implant and tooth at the uncovery visit (baseline) were set as the sources and implant at 24 hours as the sink (*P* < 0.05, Kruskal-Wallis test). Bacteria that was not trackable to the input sources are binned to the group “environment”. Boxplots not connected by same letter are significantly different

When a Bayesian modeling approach was used to estimate the source of the 24-hour peri-implant microbiome, the coverscrew chamber was identified as the primary source, contributing 62.07% ± 14.73% of the bacterial species identified (*P*< 0.05, Kruskal-Wallis). The adjoining teeth contributed 27.8% ± 10.30%, while 10% ± 10.70% of the peri-implant microbiome was traced to unknown environmental sources (potentially saliva or other oral mucosal surfaces, Fig. 1, Kruskal-Wallis, *P* < 0.005).

### Coverscrew derived pioneer species drive the dynamics of peri-implant colonization

We then interrogated the importance of these early colonizers on subsequent community dynamics using co-occurrence network analysis. Since the coverscrew chamber formed the predominant source of the 24-hour peri-implant microbiome, we designated the core coverscrew chamber bacteria (240 species) as “pioneer species”. Bacterial networks demonstrated progressively greater connectivity (with a 2-fold increase in the connections with increasingly larger hubs from uncovery to 42 weeks), indicating diversification of community synergy. Interestingly, even though the hubs expanded over time, they were anchored by the same consortia of species. Even more interesting was that most of these anchors were pioneer species (Supplementary Figures S1A-F and Supplementary Table S2). While new species were introduced into the peri-implant microbiome beginning at 24 hours, pioneer species dominated the nascent microbiome 70:30 throughout the observation period. A within-module/across-modules plot (ZiPi plot) analysis identified 15 species with a high degree of connectedness across different modules (Pi > 0.62). All 15 were pioneer species, and 13 of them belonged to the genus *Prevotella* (Supplementary Figure S2A), further attesting to the influence of pioneers on network topography.

### New species are recruited to the microbiome based on phylogenetic relatedness to pre-existing colonizers

Since the order of bacterial assembly has significant implications for colonization dynamics, we applied a mathematical model that quantifies the degree (D) to which new species inducted into a community are phylogenetically related to pre-existing community members. We discovered that implant colonization follows a significant phylogenetic underdispersion (D < 0, *P* = 0.000 5), indicating that newly introduced species are more likely to be a phylogenetic relative of resident colonizers than random recruits (Supplementary Figures S2B-C). We also discovered that the underdispersion pattern was not significant when the abundances of new or pre-existing species were inducted into the model (*P* > 0.05), indicating that the mere presence of a species is sufficient to promote colonization by a close phylogenetic relative, even if the species is present in low abundances.

We then examined the importance of pioneer species to the longitudinal acquisition of subsequent species using eLSA (Supplementary Figure S2D, *P* < 0.05, and Supplementary Table S3). 1890/3788 (50%) of edges connected pioneer species across the different time points. 128 edges were intra-generic, while 3 660 were inter-generic, suggesting a process beyond phylogenic similarity in bacterial acquisition. Furthermore, 3 414/3 660 (93.28%) of the edges were identified at uncovery, and 67.63% of these were sustained over the entire timeline of the study; pointing to a sustained effect exerted by the pioneer species. Interestingly, 94 of the 128 edges demonstrated a negative association over time. For example, the densest intra-generic association was within the genus *Prevotella*, where 66 of the relationships were negative, while 11 were positive. The negative relationship began at uncovery and was sustained throughout the 12 weeks, indicating an early niche saturation that excludes same-genera species from the concomitant acquisition.

### The peri-implant microbiome demonstrates increasing personalization over time with the inclusion of new species

Building on our previous investigations that implant surfaces exert a significantly greater selection pressure on the microbiome than host-associated factors,^13^ we calculated the level of homogeneity among subjects by computing the core microbiome at each time point at the species and sub-species/strain levels (Supplementary Figure S3). In contrast to the time of uncovery when 49% of the species were shared among all 15 individuals, only 19%−29% of the new recruits were shared among all implants in the subsequent time points, pointing to an increasing personalization of the peri-implant microbiome as it matures (Supplementary Table S4).

### The microbiome is functionally diverse across time and resilient to perturbation

Since community development is driven by shared and competing functions within an ecosystem, we inducted 17 777 functionally annotated genes into alpha- and beta-diversity analyses. Compositional Tensor Factorization revealed significant expansion of functionalities from uncovery and each subsequent time point until the placement of the final restoration two weeks after uncovery, which served to reset the functionality to baseline levels at week 3 (*P* < 0.000 1 REML test, Supplementary Figure S4A, CTF, Compositional Tensor Factorization). However, functional expansion renewed between 3 to 6 weeks and continued until 12-weeks.

At uncovery, the key functionality was transcription, and KRAB domain-containing zinc finger protein contributed predominantly to this function. Genes encoding pathways of oxidative phosphorylation (G-coupled receptor proteins, mono-oxidases, pyruvate metabolism etc.), fluid-exchange across membranes (aquaporins) were also predominant. Interestingly, at 24-hours, genes related to oxidative phosphorylation were decreased by 21%, indicating an increase in anaerobic functionality. This was also accompanied by a bloom in genes encoding replication, recombination and repair proteins, mitochondrial biogenesis, etc., consistent with community expansion through bacterial cell division (Supplementary Figure S4B). When the 1-week biome was compared to the 24-hour biome, 25 genes encoding enzyme families (hydrolases and isomerases), 20 encoding membrane transport, cellular processes and signaling were enriched (Supplementary Figure S4C, *P* < 0.05, FDR adjusted Wald test). On the other hand, 38 genes that coded for carbohydrate metabolism and enzyme families decreased in abundance (Supplementary Figure S4D, *P* < 0.05, FDR-adjusted Wald test). No significant differential abundances were evident at 3 weeks and beyond.

### Implant colonization trajectories differ from those of adjoining teeth in diversity and extent of expansion

We next compared the trajectories of functional expansion in the peri-implant and adjoining subgingival microbiomes. A similar functional volatility pattern was observed over time; however, the tooth, when compared to the implant, showed a greater functional diversity from uncovery until 12 weeks (*P* < 0.016 REML test of CTF, Supplementary Figure S5A). In corroboration, throughout all visits, a significantly different functional diversity was observed in teeth compared to implants (*P* < 0.000 1, Jaccard and Bray-Curtis indices, Supplementary Figures 5B and C, respectively). A very interesting observation was that there was an appreciable and significant decrease in functional volatility in teeth at week 3, similar to that of implants, even though the subgingival sulcus was not perturbed in the same fashion as the peri-implant crevice (i.e. placement of a restoration) (*P* < 0.001 REML test of CTF, Supplementary Figure S5A). This suggests that implant restoration might impact the subgingival microbiome and deserves further investigation.

## Discussion

Our knowledge of the early microbial colonization of the peri-implant sulcus is gleaned from studies that utilized culture-based methods, targeted microbiologic assays, and 16S rDNA sequencing.^14–20^ These studies surmised that the peri-implant sulcus is colonized within 30 minutes following implant placement,^19^ a complex flora can develop within 2 weeks,^14,15^ and that subgingival and peri-implant microbiota were similar.^14,16,18^ However, when we combined whole genome shotgun sequencing with multiple bioinformatics pipelines to longitudinally analyze the timeline for the acquisition, development, and personalization of the peri-implant microbiome from the moment the implant was uncovered through the following 12 weeks, we discovered an intricately orchestrated pattern of microbial assembly, in which the microbiota entrapped within the cover screw chamber play a key role.

Our principal finding was the importance of the coverscrew chamber microbiome in influencing peri-implant colonization. The first evidence was that the coverscrew chamber hosts a commensal-rich, diverse microbial community.^21^ While it is possible that these organsims were derived from saliva or other teeth, one notable observation was that the coverscrew chamber microbiome was homogeneous among all the subjects, suggesting that the implant material itseld might play an important role in modulating the flora, building a unique peri-implant niche. This lends credence to Becking and Beijerinck’s famous statement that “everything is everywhere, but the environment selects”.^22^ The second finding was the coverscrew chamber microbiota served as pioneer species. Pioneer species have adaptation abilities that help them colonize a habitat, and they grow fast.^23–25^ Evidence from non-human ecosystems shows that pioneers exploit the environmental changes that occur in newly created habitats (primary succession) or in recently disturbed environments (secondary succession) to influence community structure and composition.^23,24^ In line with this, we discovered that peri-implant pioneers exert an early and sustained effect as network influencers and controllers of resources. It was interesting to note that the peri-implant microbiome maintained a similar network topography in the face of progressive personalization and expansion; and that the hubs were predominantly anchored by the pioneers derived from the cover screw despite the “age” and idiosynchonization of the peri-implant community. The stable colonization by the pioneers, and their ability to influence acquisition might also serve to explain the resilience of this community to perturbation (such as that imposed by crown placement).

Another key finding was that bacterial succession in the peri-implant community follows an under-dispersion pattern of species recruitment. This implies that species are most likely to be recruited to this community if an evolutionarily close neighbor is already present.^26,27^ Interestingly, the mere presence of a ‘close neighbor’ is sufficient to promote colonization, suggesting that pioneer species can make the environment more favorable for a phylogenetic neighbor to colonize, irrespective of their abundance.^26^ This finding is particularly significant in patients with periodontitis, as previously shown, pathogens can translocate from the affected tooth into the cover screw chamber during implant placement.^28^ This pathogen-rich community can then seed the pristine peri-implant sulcus, creating a virulent microbiome from inception. This corroborates as a possible explanation for the higher risk of peri-implant diseases in subjects with untreated or poorly-controlled periodontitis.^9^

In nature, bacterial succession is triggered by optimal ecological restoration, facilitative interactions, and subsequent competition.^29–31^ The accepted mechanism is that facilitative interactions occur between distantly-related species while competition occurs between closely-related species.^29^ Corroborating this, we observed robust and statistically significant negative correlations between members of the genus *Prevotella* throughout the colonization process. Within the implant community, we observed strong negative correlations between numerically dominant species with numerically rare taxa, and positive correlations between numerically rare taxa.

In the present investigation, we discovered that shared functionality is an important determinant of bacterial succession; and that the functions encoded by the pioneers create an environment favorable for cell replication and biofilm maturation. Maturation is accompanied by a progressive increase in anaerobiosis from weeks 1-3. However, the predominant functionalities continue to be largely oxygen-dependent, namely, enzyme families, nucleotide metabolism, lipid metabolism, and energy metabolism. It is known that the functions expressed by members of a microbial community are impacted by their neighbors and by the physiological features of their environment;^32,33^ however, it was not within the scope of this study to investigate spatial relationships of the colonizers. Further studies that map the biogeography of the peri-implant sulcus are warranted to localize these interactions within the biofilm communities.

One final notable observation was that the colonization trajectories of implants differed from those of adjoining teeth, corroborating and building on our previous studies which have shown that the peri-implant biome is different from the tooth biome.^12,34^ Further, an interesting and significant influence was observed in the subgingival biome following the placement of an implant crown, suggesting that implant restoration might impact the subgingival microbiome, which deserves further investigation.

The present investigation used 15 subjects with a set of very stringent inclusion criteria. While this decreased the variability associated with clinical studies, it also decreased the generalizability of our data to other populations undergoing dental implants therapy (e.g., smokers, e-cigarette users, uncontrolled or poorly-controlled diabetics), as well as other implant systems, materials and surfaces. We also used a two-stage protocol; therefore, the findings cannnot be extrapolated to one-stage or one-piece implants. The two-stage protocol also limited our ability to track the sources of microbiota within the cover screw chamber at the time of implant placement. It is possible that saliva, the adjoining tooth, the adjacent subgingival crevice and the mucosa were all sources of the coverscrew bacteria at the time of implant placement. However, the implants were carefully isolated during second-stage surgery and cover screw retrieval.

Furthermore, to address potential biases in microbiome studies, we implemented several strategies: (a) all samples were collected by a single trained dentist using a standardized procedure, including supragingival plaque removal, isolation with cotton rolls, and consistent timing; (b) contamination was minimized during sample collection, processing, and DNA isolation; (c) the core metagenome was analyzed to reduce the influence of allochthonous species and genes, following an approach adapted from the Human Microbiome Project; (d) and PCR and sequencing artifacts were controlled by incorporating positive and negative controls.

In summary, within the study’s limits, we find evidence to support progressive personalization and expansion of the peri-implant microbiome over 12 weeks. Our data underscores the importance of pre-existing species in the oral environment during implant placement and highlights the importance of placing implants in periodontally healthy individuals or following the successful resolution of periodontal disease.

## Material and methods

### Ethics statement

This was a single-center, longitudinal cohort study approved by The Ohio State Institutional Review Board (2016H0134) and carried out according to the approved protocol. The study conformed to the STROBE guidelines for observational studies. All participants gave written informed consent prior to enrollment.

### Subject selection

We recruited systemically [ASA 1 (American Society of Anesthesiologists Physical Status Classification I)] and periodontally healthy individuals [attachment loss ≤ 1 mm; less than three sites with 4 mm of probe depths (PD); bleeding index (BOP) ≤ 20%]. Inclusion criteria included partially edentulous individuals ≥ 21 years of age who were scheduled to receive a single, tooth-bounded tapered screw-vent (TSV) implant with microtextured surface as a part of a two-stage protocol. Exclusion criteria consisted of being a current or former smoker (defined as those who had smoked more than 100 cigarettes in their lifetime) or e-cigarette user, controlled or uncontrolled diabetes, HIV infection, use of immunosuppressant medications, bisphosphonates or steroids, antibiotic therapy or oral prophylactic procedures within the preceding 3 months, requirement for antibiotic prophylaxis before dental examinations, and fewer than 20 teeth in the dentition.

### Sample size estimation

We based our sample size estimation on our peri-implant dual transcriptomic study,^11^ experimental gingivitis studies^35,36^ and longitudinal transcriptional analysis of the periodontitis.^37^ In a typical representation of the peri-implant transcriptome, the transcripts are categorized by approximately 20 pathways. In our power calculations, we took a slightly conservative approach and assumed we would look at approximately 25 pathways in this data and will need to adjust for multiplicity using the Holm-Bonferroni method, which assures an experiment family-wise error rate of less than 5% if we use a critical value of 0.002 = 0.05/25 on individual analyses. By interrogating data from 15 subjects over 6-time intervals, we anticipated that we would be able to detect a contrast in the transformed scale of 1.59 degrees for the row (baseline versus each visit) effects.

### Study design

**Fig. 2:**
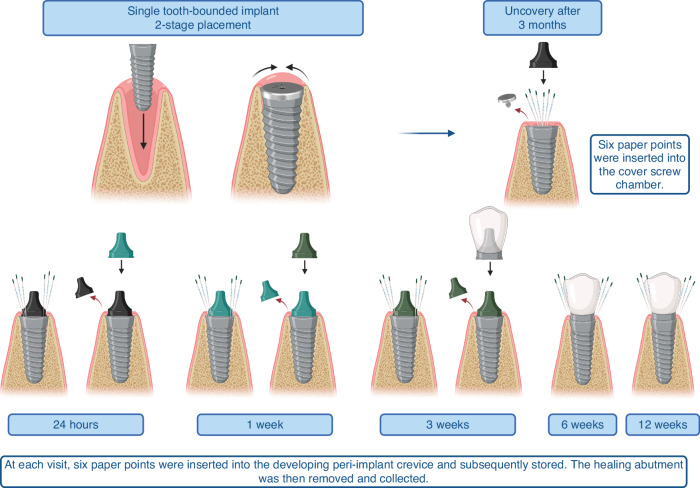
Clinical study design

Briefly, under a sterile and controlled surgical procedure and after extra- and intra-oral asepsis, the surgical area was anaesthetized, a modified crestal incision was made and a full thickness mucoperiosteal flap was raised. Implants were placed at bone level, and covered with a 0 mm healing abutment (also known as a cover screw), and the overlying mucosa was secured by primary closure (Fig. 2). The implants were surgically uncovered 3 months later under isolation with sterile cotton swabs, and the cover screw was removed. At the uncovery visit, six sterile endodontic paper points (Caulk-Dentsply, Milford, DE, USA) were inserted into the cover screw chamber for 30 seconds and stored. The cover screw was replaced by a transmucosal healing abutment. Indices of periodontal health (gingival index –GI,^38^ plaque index – PI,^39^ and bleeding on probing - BOP) were recorded on the adjoining teeth. Baseline biofilm samples were collected from the adjoining teeth by inserting six paper points into the gingival sulcus of adjoining teeth. The adjoining teeth were scaled, polished, and flossed to remove adherent biofilm and monitor de novo colonization of the teeth’s subgingival microbiome simultaneously with the implant. Following this baseline visit, the participants were seen for 5 more visits (24 hours, 1 week, 3 weeks, 6 weeks, and 12 weeks). At each visit, six paper points were inserted into the developing peri-implant crevice and subsequently stored. The healing abutment was then removed and collected. A new abutment was placed at the end of each visit. Subjects were seen for placement of a final restoration after two weeks, following which the protocol continued in a similar fashion.

### Sample collection

Prior to sample collection, selected sites were isolated with cotton rolls, and supragingival plaque was removed. Subgingival and submucosal biofilm samples were collected from each patient every visit by inserting six sterile endodontic paper points into the peri-implant and the adjoining periodontal sulci for 30 seconds. The samples were then separately placed in two 1.5 mL microcentrifuge tubes containing 100 μL of RNA*Later*. Samples were frozen at 80 °C until further analysis.

### DNA isolation and sequencing

Bacteria were separated from the paper points by adding 200ul of phosphate-buffered saline to the tubes and vortexing. The paper points were then removed, and DNA was isolated with a Qiagen DNA MiniAmp kit (Qiagen, Valencia, CA, USA), following the protocol according to the manufacturer’s instructions. Libraries were generated using the NEBNext® UltraTM II FS DNA Library Preparation Kit for Illumina (New England Biolabs, Inc). 100 ng of the sample was used as input. DNA was fragmented to 100 – 250 bp. Adapters were then ligated to the fragments. 12 unique adapters were used so that 12 samples could be pooled together during sequencing. Size selection was not performed; however, the adapter-ligated samples were cleaned before PCR enrichment. 6 cycles of PCR were completed before cleaning and pooling the samples. Pooled libraries were sequenced on the Illumina HiSeq4000 platform using 150 bp paired-end chemistry.

### Metagenomic data processing and statistical analysis

#### Metagenomic sequence analysis

Sequences were quality filtered with Sickle and screened for human DNA with Bowtie 2.^40^ Phylogenetic assignment was performed using Woltka and Bowtie 2 mapping to the Human Oral Microbiome Database (HOMD).^41^ Prodigal was used for coding sequence (CDS) prediction, and genes were aligned against the NCBI nonredundant database of proteins using DIAMOND.^42^ Biological pathways and protein functional categories were determined by annotation to the Kyoto Encyclopedia of Genes and Genomes orthology (KEGG)^43^ using MEGAN.^44^ Bioinformatic analysis was performed using the QIIME 2 2023.5^45^ and PhyloToAST v1.4.^46^ Alpha diversity was evaluated through calculation of the Shannon index^47^ and significance of longitudinal differences through repeated measures ANOVA. Longitudinal differences in beta diversity were computed using the Jacquard and Bray-Curtis indices within the Compositional Tensor Factorization as part of the TEMPoral TEnsor Decomposition (TEMPTED) analysis framework.^48,49^ Linear Discriminant Analysis was used for dimensionality reduction and ordination. The Bayesian analysis-based SourceTracker^50^ was used to identify probable sources of the peri-implant sulcus microbiome.

Colonization dynamics over time were ere examined using (1) Network analysis: Sparse Co-occurrence Network Investigation for Compositional Data (SparCC) to compute species-level co-occurrence networks for each time point (*r* > 0.80). A correlation network was built, and modules were detected using SCNIC.^51^ Network graphs were visualized in Gephi,^52^ and within-module/across-modules analysis was performed using the built-in tools. (2) Extended Local Similarity Analysis (eLSA): was used to identify the longitudinal effect of the species on the acquisition of subsequent species, which examines the co-occurrence (edge) of species X on Species Y across different time points, while reporting both the strength of the relationship, and the timepoint when Species X started affecting species Y, and whether there is a delay in this effect.^53^ (3) Phylogenetic model for species recruitment: This model, described by Darcy et al.,^27^ tests whether new species are more or less likely to be recruited into the community in the presence of a close phylogenetic relative (nepotism) or if the process was agnostic to evolutionary relationships (neutral model). The model estimates an empirical dispersion parameter “*D”*, which quantifies the degree to which first-time species are phylogenetically related to the pre-existing community residents. If D ≠ 0, then species are preferentially added if they have relatively close (D < 0) or relatively far (D > 0) phylogenetic distance to the resident community.

Differential abundance analysis of functional genes was carried out using DESeq2 and p-values adjusted for multiple testing (false discovery rate [FDR] < 0.05, FDR-adjusted Wald test).^54^ The results were used to construct KEGG maps using the web-based Interactive Pathways Explorer (iPath3).^54^ Statistical analysis for longitudinal and within-site comparisons was conducted using JMP software using the restricted maximum likelihood method (REML) to reduce biases in variance and co-variance.

Sequences are deposited in the NCBI Sequence Read Archive under the project ID PRJNA1082341.

## Supplementary information

Figure S1A-F: Community dynamics during the development of the peri-implant sulcus

Supplementary Figure 2: Recruitment of species follows principles of nepotism while pioneer species impact recruitment of new species and flow of resources

Supplementary Figure 3. Waterfall plot of the core microbiome

Supplementary Figure 4. Functional dynamics of the developing peri-implant microbiome demonstrates stability after 3 weeks

Supplementary Figure 5: Implant colonization trajectories differ from those of adjoining teeth in diversity and extent of expansion

Supplementary Table 3: Data supporting Figure 4 D - Extended local similarity analysis

Supplementary Table 1:

Supplementary Table 2:

Supplementary Table 4. Data supporting Figure 5 - Microbiome core, 100%

Supplementary Table and Figure Legends

## Data Availability

The sequence materials have been uploaded to the Sequence Read Archives (SRA) of the National Center for Biotechnology Information (NCBI) database with the following identifier: PRJNA1082341.

## References

[1] NIDCR. Tooth Loss Among Adults 20−64. http://www.nidcr.nih.gov/DataStatistics/FindDataByTopic/ToothLoss/ToothLossAdults20to64.htm (2014).

[2] Emami, E., De Souza, R. F., Kabawat M. & Feine J. S. The impact of edentulism on oral and general health. *Int. J. Dentistry*. **2013**, 1−7 (2013). 10.1155/2013/498305 PMC3664508 23737789

[3] Jain, N., Dutt, U., Radenkov, I. & Jain S. WHO’s global oral health status report 2022: Actions, discussion and implementation. *Oral Dis*. 30, 73–79 (2023). 10.1111/odi.14516 36680388

[4] Brånemark, P. I. Osseointegration and its experimental background. *J. Prosthet. Dent.* **50**, 399–410 (1983). 6352924 10.1016/s0022-3913(83)80101-2

[5] Becker, W., Becker, B. E., Newman, M. G. & Nyman, S. Clinical and microbiologic findings that may contribute to dental implant failure. *Int J. Oral. Maxillofac. Implants* **5**, 31–38 (1990). 2391137

[6] Dreyer, H. et al. Epidemiology and risk factors of peri-implantitis: A systematic review. *J. Periodontal Res.* **53**, 657–681 (2018). 29882313 10.1111/jre.12562

[7] Esposito, M., Grusovin, M. G. & Worthington, H. V. Interventions for replacing missing teeth: treatment of peri-implantitis. *Cochrane Database Syst. Rev.* **1**, CD004970 (2012). 22258958 10.1002/14651858.CD004970.pub5 PMC6786958

[8] Berglundh, T. et al. Peri-implant diseases and conditions: Consensus report of workgroup 4 of the 2017 World Workshop on the Classification of Periodontal and Peri-Implant Diseases and Conditions. *J. Clin. Periodontol.* **45**, S286–S291 (2018). 29926491 10.1111/jcpe.12957

[9] Romandini, M. et al. Prevalence and risk/protective indicators of peri-implant diseases: A university-representative cross-sectional study. *Clin. Oral. Implants Res.* **32**, 112–122 (2021). 33210772 10.1111/clr.13684

[10] Belibasakis, G. N. & Manoil, D. Microbial community-driven etiopathogenesis of peri-implantitis. *J. Dental Res.* 100, 21–28 (2021). 10.1177/0022034520949851 PMC7754824 32783779

[11] Ganesan, S. M. et al. Biome-microbiome interactions in peri-implantitis: A pilot investigation. *J. Periodontol.* **93**, 814–823 (2022). 35073418 10.1002/JPER.21-0423 PMC9187590

[12] Dabdoub, S. M., Tsigarida, A. A. & Kumar P. S. Patient-specific analysis of periodontal and peri-implant microbiomes. *J. Dent. Res.* **92**, 168S–75S (2013). 10.1177/0022034513504950 PMC3827621 24158341

[13] Sinjab, K. et al. Impact of surface characteristics on the peri-implant microbiome in health and disease. *J. Periodontol.* **95**, 244−255 (2024). 10.1002/JPER.23-0205 PMC10909931 37665015

[14] Quirynen, M. et al. Dynamics of initial subgingival colonization of ‘pristine’ peri-implant pockets. *Clin. Oral. Implants Res.* **17**, 25–37 (2006). 16441782 10.1111/j.1600-0501.2005.01194.x

[15] Quirynen, M. et al. Initial subgingival colonization of ‘pristine’ pockets. 2005. 10.1177/154405910508400409 15790740

[16] Payne, J. B. et al. Subgingival microbiome colonization and cytokine production during early dental implant healing. *mSphere*. **2**, e00527−17 (2017). 10.1128/mSphereDirect.00527-17 PMC5705808 29202047

[17] Van Winkelhoff, A. J., Goené, R. J., Benschop, C. & Folmer, T. Early colonization of dental implants by putative periodontal pathogens in partially edentulous patients. *Clin. Oral. Implants Res.* **11**, 511–520 (2000). 11168244 10.1034/j.1600-0501.2000.011006511.x

[18] de Freitas, A. R. et al. Oral bacterial colonization on dental implants restored with titanium or zirconia abutments: 6-month follow-up. *Clin. Oral. Investig.* **22**, 2335–2343 (2018). 29349504 10.1007/s00784-018-2334-0

[19] Fürst, M. M., Salvi, G. E., Lang, N. P. & Persson, G. R. Bacterial colonization immediately after installation on oral titanium implants. *Clin. Oral. Implants Res.* **18**, 501–508 (2007). 17501978 10.1111/j.1600-0501.2007.01381.x

[20] Silva-Boghossian, C. M., Duarte, P. T., Silva DG, da, Lourenço, T. G. B. & Colombo, A. P. V. Colonization dynamics of subgingival microbiota in recently installed dental implants compared to healthy teeth in the same individual: a 6-month prospective observational study. *J. Appl. Oral Sci*. **31**, 1−10 (2023). 10.1590/1678-7757-2023-0134 PMC10519670 37729258

[21] Avila, M., Ojcius, D. M. & Zlem, Y. Ö. The oral microbiota: living with a permanent guest. *DNA Cell Biol*. **28**, 405–411 (2009). 10.1089/dna.2009.0874 PMC2768665 19485767

[22] De Wit, R. & Bouvier, T. ‘Everything is everywhere, but, the environment selects’; what did Baas Becking and Beijerinck really say? *Environ. Microbiol*. **8**, 755–758 (2006). 10.1111/j.1462-2920.2006.01017.x 16584487

[23] Holmberg, T. J. *BIOL 1213*. LibreTexts; Northwestern Connecticut Community College https://bio.libretexts.org/@go/page/79240 (2022). Retrieved from https://bio.libretexts.org/Sandboxes/tholmberg_at_nwcc.edu/BIOL_1213

[24] Clark, M. A., Douglas, M. & Choi, J. *Biology 2e*. 2nd edn. Vol. 1. (OpenStax; 2018).

[25] Sottosanti, K. ‘pioneer species’. In: *Encyclopedia Britannica*. https://www.britannica.com/science/pioneer-species (2023).

[26] Darwin, C. *On the Origin of Species* (Murray, 1859).

[27] Darcy, J. L. et al. A phylogenetic model for the recruitment of species into microbial communities and application to studies of the human microbiome. *ISME J.* **14**, 1359–1368 (2020). 32076128 10.1038/s41396-020-0613-7 PMC7242462

[28] Sumida, S., Kazuyuki, I., Kishi, M. & Okuda, K. Transmission of periodontal disease-associated bacteria from teeth to osseointegrated implant Regions. **17**, 696–702 (2002). 12381070

[29] Verdú, M., Gómez-Aparicio, L. & Valiente-Banuet, A. Phylogenetic relatedness as a tool in restoration ecology: A meta-analysis. *Proc. R. Soc. B: Biol. Sci.* **279**, 1761–1767 (2012). 10.1098/rspb.2011.2268 PMC3297466 22158955

[30] Connell, J. H. & Slatyer, R. O. Mechanisms of succession in natural communities and their role in community stability and organization. *American Naturalist*. 111, 1119−1144 (1977).

[31] Castillo, J. P., Verdú, M. & Valiente-Banuet, A. Neighborhood phylodiversity affects plant performance. *Ecology* **91**, 3656–3663 (2010). 21302836 10.1890/10-0720.1

[32] Welch, J. L. M., Rossetti, B. J., Rieken, C. W., Dewhirst, F. E. & Borisy, G. G. Biogeography of a human oral microbiome at the micron scale. *Proc. Natl Acad. Sci. USA.* **113**, E791–E800 (2016). 26811460 10.1073/pnas.1522149113 PMC4760785

[33] Welch, J. L. M., Hasegawa, Y., McNulty, N. P., Gordon, J. I. & Borisy, G. G. Spatial organization of a model 15-member human gut microbiota established in gnotobiotic mice. *Proc. Natl Acad. Sci. USA.* **114**, E9105–E9114 (2017). 29073107 10.1073/pnas.1711596114 PMC5664539

[34] Kumar, P. S., Mason, M. R., Brooker, M. R. & O’Brien, K. Pyrosequencing reveals unique microbial signatures associated with healthy and failing dental implants. *J. Clin. Periodontol.* **39**, 425–433 (2012). 22417294 10.1111/j.1600-051X.2012.01856.x PMC3323747

[35] Matthews, C. R., Joshi, V., de Jager, M., Aspiras, M. & Kumar, P. S. Host-bacterial interactions during induction and resolution of experimental gingivitis in current smokers. *J. Periodontol.* **84**, 32–40 (2013). 22420875 10.1902/jop.2012.110662

[36] Joshi, V. et al. Smoking decreases structural and functional resilience in the subgingival ecosystem. *J. Clin. Periodontol.* **41**, 1037–1047 (2014). 25139209 10.1111/jcpe.12300

[37] Duran-Pinedo, A. et al. Long-term dynamics of the human oral microbiome during clinical disease progression. *BMC Biol*. **19**, 2−17 (2021). 10.1186/s12915-021-01169-z PMC8572441 34742306

[38] Loe, H. & Silness, J. Periodontal disease in pregnancy.I. Prevalence and severity. *Acta Odontol. Scand.* **21**, 533–551 (1963). 14121956 10.3109/00016356309011240

[39] Silness, J. & Loe, H. Periodontal disease in pregnancy. II. Correlation between oral hygiene and periodontal condition. *Acta Odontol. Scand.* **22**, 121–135 (1964). 14158464 10.3109/00016356408993968

[40] Langmead, B. & Salzberg, S. L. Fast gapped-read alignment with Bowtie 2. *Nat. Methods* **9**, 357–359 (2012). 22388286 10.1038/nmeth.1923 PMC3322381

[41] Escapa, I. F. et al. New Insights into Human Nostril Microbiome from the Expanded Human Oral Microbiome Database (eHOMD): a Resource for the Microbiome of the Human Aerodigestive Tract. *mSystems*. **3**, 1−20 (2018). 10.1128/mSystems.00187-18 PMC6280432 30534599

[42] Buchfink, B., Xie, C. & Huson, D. H. Fast and sensitive protein alignment using DIAMOND. *Nat. Methods* **12**, 59–60 (2015). 25402007 10.1038/nmeth.3176

[43] Kanehisa, M. Goto S. KEGG: kyoto encyclopedia of genes and genomes. *Nucleic Acids Res.* **28**, 27–30 (2000). 10592173 10.1093/nar/28.1.27 PMC102409

[44] Huson, D. H., Auch, A. F., Qi, J. & Schuster, S. C. MEGAN analysis of metagenomic data. *Genome Res.* **17**, 377–386 (2007). 17255551 10.1101/gr.5969107 PMC1800929

[45] Bolyen, E. et al. Reproducible, interactive, scalable and extensible microbiome data science using QIIME 2. *Nat. Biotechnol*. **37**, 852–857 (2019). 10.1038/s41587-019-0209-9 PMC7015180 31341288

[46] Dabdoub, S. M. et al. PhyloToAST: Bioinformatics tools for species-level analysis and visualization of complex microbial datasets. *Sci. Rep*. **6**, 1−9 (2016). 10.1038/srep29123 PMC4928119 27357721

[47] Spellerberg, I. F. & Fedor, P. J. A tribute to Claude Shannon (1916–2001) and a plea for more rigorous use of species richness, species diversity and the ‘Shannon–Wiener’ Index. *Glob. Ecol. Biogeogr.* **12**, 177–179 (2003).

[48] Han, R., Shi, P. & Zhang, A. R. Guaranteed functional tensor singular value decomposition. *J. Am. Stat. Assoc.* **119**, 995–1007 (2023). 10.1080/01621459.2022.2153689 PMC11267031 39055126

[49] Shi, P., Martino, C., Han, R. et al. TEMPTED: time-informed dimensionality reduction for longitudinal microbiome studies. *Genome. Biol*. **25**, 317 (2024). 10.1186/s13059-024-03453-x PMC11657513 39696594

[50] McGhee, J. J. et al*.* Meta-SourceTracker: Application of Bayesian source tracking to shotgun metagenomics. *PeerJ*. **8**, 2−18 (2020). 10.7717/peerj.8783 PMC7100590 32231882

[51] Shaffer, M., Thurimella, K., Sterrett, J. D. & Lozupone, C. A. SCNIC: Sparse correlation network investigation for compositional data. *Mol. Ecol. Resour.* **23**, 312–325 (2023). 36001047 10.1111/1755-0998.13704 PMC9744196

[52] Bastian, M., Heymann, S., & Jacomy, M. (2009). Gephi: an open source software for exploring and manipulating networks. In *Proc. International AAAI Conference on Web and Social Media*, 3(1), 361−362. 10.1609/icwsm.v3i1.13937.

[53] Xia, L. C. et al. Extended local similarity analysis (eLSA) of microbial community and other time series data with replicates. *BMC Syst. Biol*. **5**, 1−12 (2011). 10.1186/1752-0509-5-S2-S15 PMC3287481 22784572

[54] Darzi, Y., Letunic, I., Bork, P. & Yamada, T. iPath3.0: interactive pathways explorer v3. *Nucleic Acids Res.* **46**, W510–W513 (2018). 29718427 10.1093/nar/gky299 PMC6031023

