# Supplementary figures and images for: Community dynamics during de novo colonization of the nascent peri-implant sulcus
Source: Int J Oral Sci. 2025 Apr 29;17:37. doi: 10.1038/s41368-025-00367-7 (PMC12041454; doi:10.1038/s41368-025-00367-7)

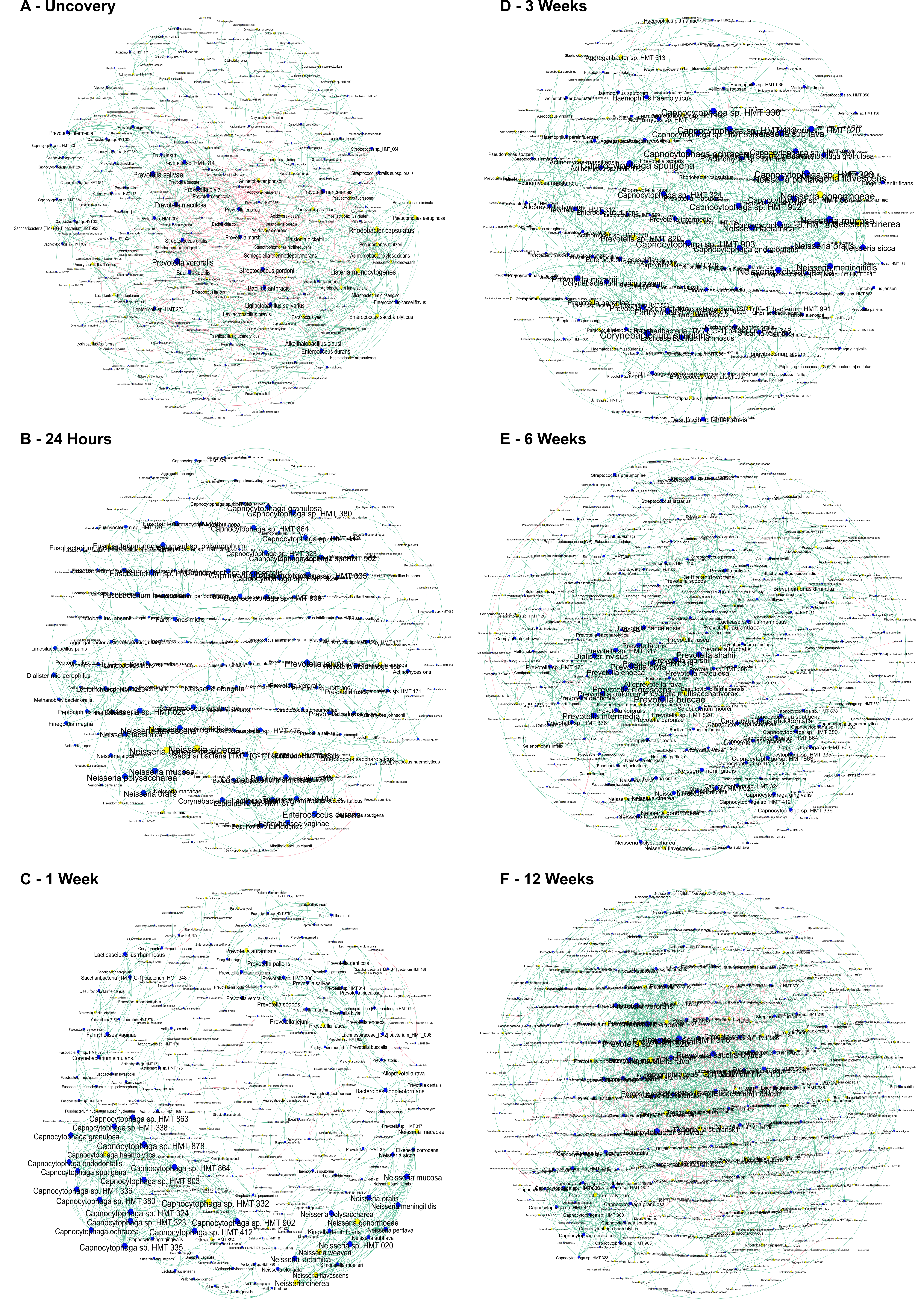

Supplement: Supplementary file 1 — Figure S1A-F: Community dynamics during the development of the peri-implant sulcus [file 41368_2025_367_MOESM1_ESM.png]

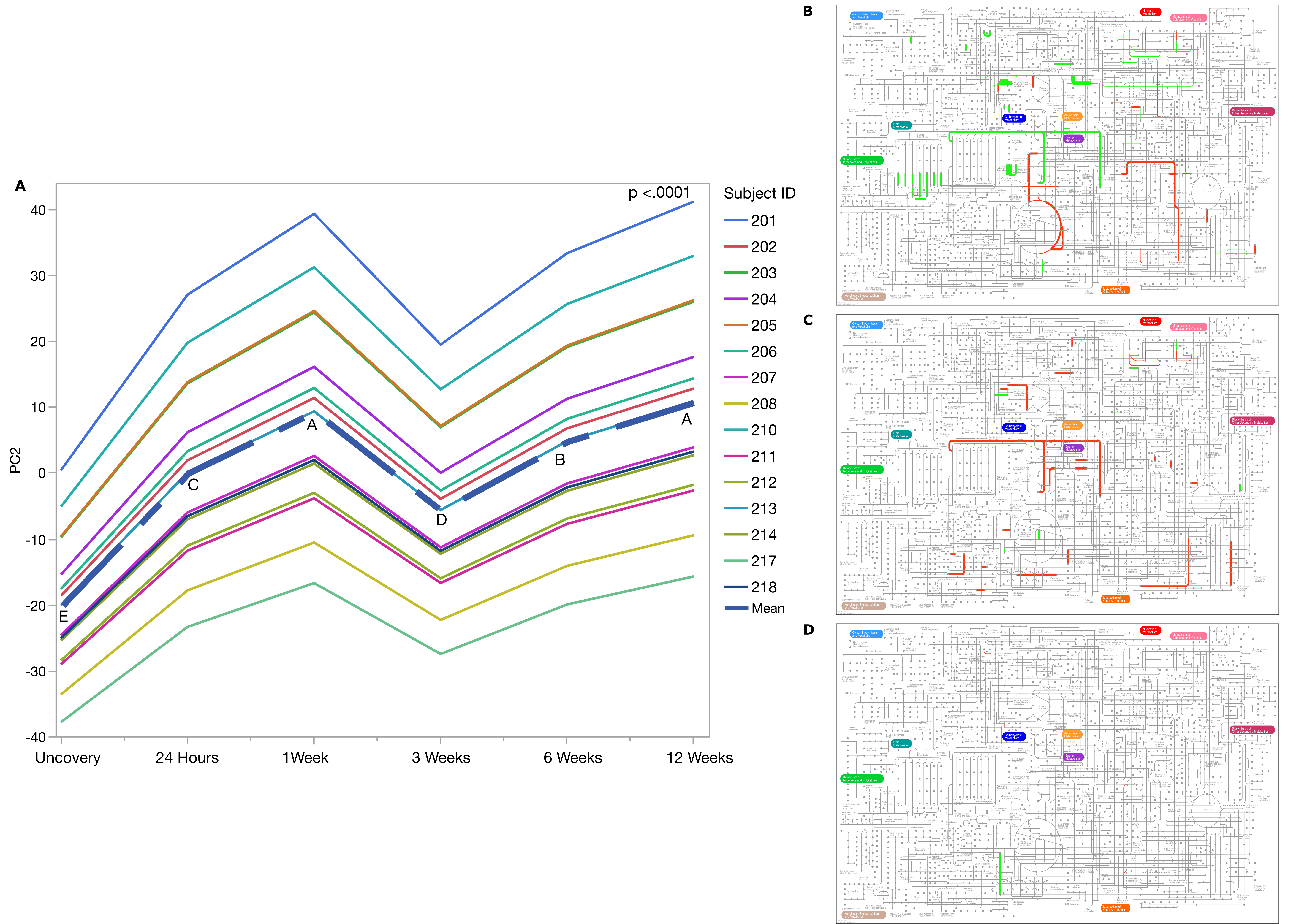

Supplement: Supplementary file 4 — Supplementary Figure 4. Functional dynamics of the developing peri-implant microbiome demonstrates stability after 3 weeks [file 41368_2025_367_MOESM4_ESM.png]

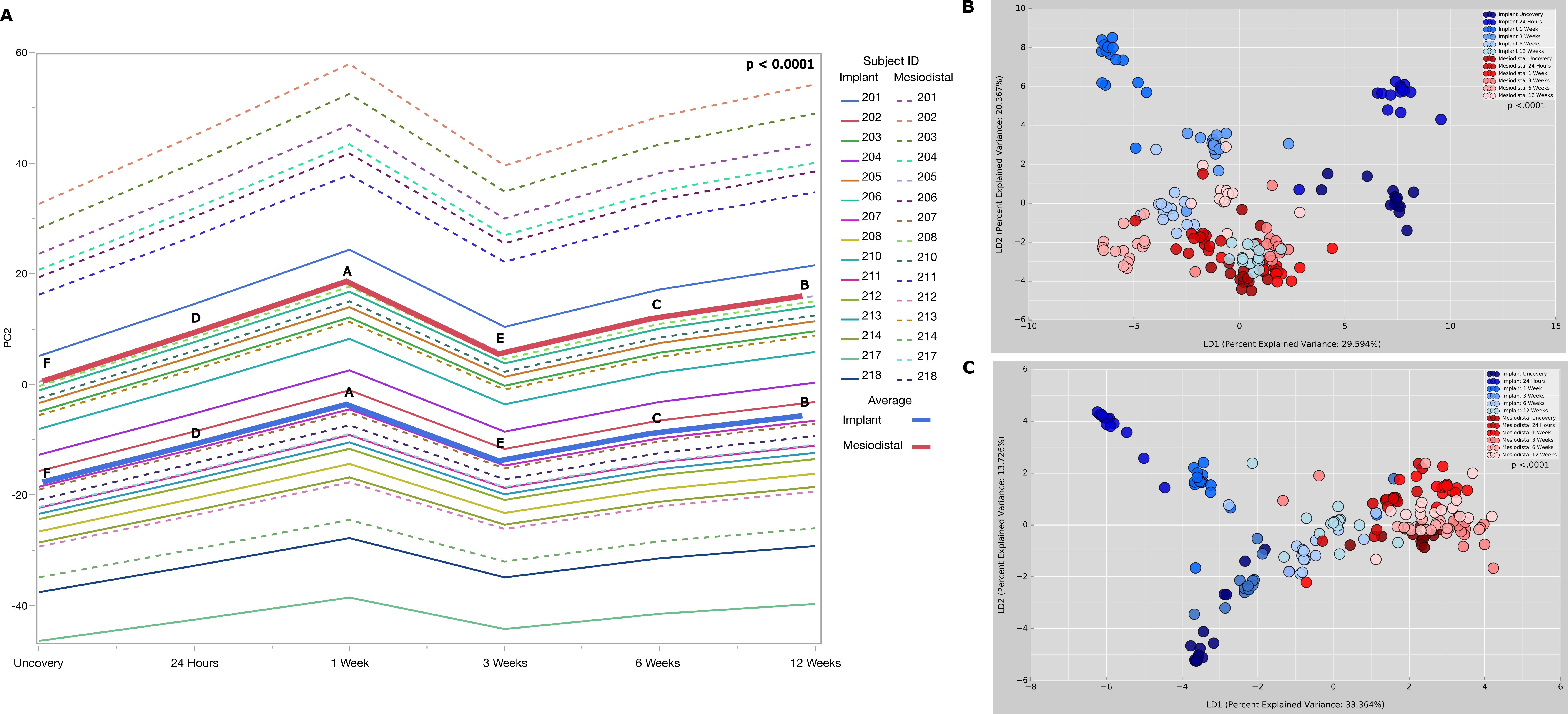

Supplement: Supplementary file 5 — Supplementary Figure 5: Implant colonization trajectories differ from those of adjoining teeth in diversity and extent of expansion [file 41368_2025_367_MOESM5_ESM.png]
