# Supplementary material for: Community dynamics during de novo colonization of the nascent peri-implant sulcus
Source: Int J Oral Sci. 2025 Apr 29;17:37. doi: 10.1038/s41368-025-00367-7 (PMC12041454; doi:10.1038/s41368-025-00367-7)
Supplement: Supplementary file 10 — Supplementary Table and Figure Legends [file 41368_2025_367_MOESM10_ESM.docx]

**Supplementary Materials**

**Supplementary Table 1:** Data supporting Figure 2 A – Coverscrew chamber microbiome.

**Supplementary Table 2:** Data supporting Figure 3 A-F - Sparse Co-occurrence Network Investigation for Compositional data. Species colored in blue are pioneers.

**Supplementary Table 3:** Data supporting Figure 4 D - Extended local similarity analysis.

**Supplementary Table 4.** Data supporting Figure 5 - Microbiome core, 100%

**Supplementary Figure 1:Community dynamics during the development of the peri-implant sulcus.** Network graphs of implant at baseline, 24 hours, 1 week, 3 weeks, 6 weeks, and 12 weeks based on Sparse Co-occurrence analysis are shown in panels A-F. Each network graph contains nodes (circles sized by relative abundance per group) and edges (lines). Nodes colored in blue represent the pioneer species, while yellow nodes represent new species. Green edges represent positive correlation, while red edges represent negative correlation (r≥ |0.80|). Data supporting this figure can be found in Supplementary Material Table S2.

**Supplementary Figure 2: Recruitment of species follows principles of nepotism while pioneer species impact recruitment of new species and flow of resources.** Within-module/across modules plot (ZiPi plot) analysis of the nodes following module identification with SCNIC(Pi  > 0.62) are shown in Panel A. Panel B demonstrates empirical (dashed) and surrogate phylodiversity accumulation over the 12 week observation period. Surrogate curves are colored according to the dispersion (D) value. New species with a previously detected close relative are shown in blue, while those that do not have a close relative are shown in green. The surrogate curve (teal) is above the empirical curve (dashed), indicating that species recruitment is phylogenetically constrained, and follows nepotism. Panel C represents the dispersion parameter (D) estimates as a violin plot. The dot in the center of a violin is the mean, and bars represent 95% confidence intervals for the D estimate. Panel D represents a Sankey diagram of the Extended local similarity analysis (eLSA) revealing associations between pioneers and non-pioneer species over 12 weeks. Data supporting this figure can be found in Supplementary Material Table S3.

**Supplementary Figure 3.** Is a waterfall plot of the core microbiome (species present in ≥100% of individuals) over time. Each bar indicates the presence of a species at the particular time point.

**Supplementary Figure 4. Functional dynamics of the developing peri-implant microbiome demonstrates stability after 3 weeks.** Panel A shows β-diversity across time, as estimated by Compositional Tensor Factorization of functional genes. There was a significant increase in functional diversity between baseline and 3-weeks, followed by functional stabilization (p <0.0001 REML test). Panels B-D represent functional pathways that were diiferentially abundant during the 12-week observation period (P<0.05, FDR-adjusted Wald Test). Line thickness is sized by log(2) fold change. Panel B: Differences between uncovery and 24 hours. Panel C: Differences between 24 hours and 1 week. Panel D: Differences between 1- and 3-weeks. No signifncat differences were detected after 3 weeks.

**Supplementary Figure 5: Implant colonization trajectories differ from those of adjoining teeth in diversity and extent of expansion.** Panel A shows β-diversity across time, as estimated by Compositional Tensor Factorization of functional genes (Location: Implants versus Adjoining Teeth, p <0.016; Visits: p <0.0001; Visit*Location: p <0.0001, REML test). Panel B shows Linear discriminant analysis (LDA) of Jaccard index clustered by site (implant and teeth) and visit, The microbiome demonstrated significant differences based on both, site type and time (p <0.0001 REML test). Panel C shows Linear discriminant analysis (LDA) of Bray-Curtis distances clustered by site (implant and teeth) and visit. The microbiome demonstrated significant differences based on both, site type and time (p <0.0001 REML test).
